# Correlation between CT images of lateral plateau and lateral meniscus injuries in patients with Schatzker II tibial plateau fractures:a retrospective study

**DOI:** 10.1186/s12891-021-04967-2

**Published:** 2022-01-03

**Authors:** Ying Pu, Zhu Lei, Ding Wenge, Xu Yue, Jiang Xiaowei, Wang Kejie, Zhao Yiwen, Huang Zhihui, Dai Xiaoyu

**Affiliations:** 1 Department of Orthopedics, Changshu Hospital Affiliated to Nanjing University of Chinese Medicine, 6 Huanghe Road, Changshu, Jiangsu Province, 215500 China; 2 Department of Orthopedics, The Third Affiliated Hospital of Soochow University, 185 Juqian Road, Changzhou, Jiangsu Province, 213000 China

**Keywords:** Tibial plateau fractures, Lateral meniscus injuries, Lateral plateau depression, Lateral plateau widening, CT

## Abstract

**Background:**

There is a great deal of controversy on whether routine MRI examination is needed for fresh fractures while the vast majority of patients with tibial plateau fractures (TPFs) receive preoperative X-ray and CT examinations. The purpose of the study was to analyze the exact correlation between CT images of lateral plateau and lateral meniscus injuries in Schatzker II TPFs.

**Methods:**

A total of 296 patients with Schatzker II TPFs from August 2012 to January 2021 in two trauma centers were enrolled for the analysis. According to the actual situation during open reduction internal fixation (ORIF) and knee arthroscopic surgery, patients were divided into meniscus injury (including rupture, incarceration, etc.) and non-meniscus injury groups. The values of both lateral plateau depression (LPD) and lateral plateau widening (LPW) of lateral tibial plateau on CT images were measured, and their correlation with lateral meniscus injury was then analyzed. The relevant receiver operating characteristic (ROC) curve was drawn to evaluate the optimal cut-off point of the two indicators which could predict meniscus injury.

**Results:**

The intra- and inter-observer reliabilities of LPD and LPW were acceptable (intraclass correlation coefficient (ICC) > 0.8). The average LPD was 13.2 ± 3.2 mm while the average value of the group without meniscus injury was 9.4 ± 3.2 mm. The difference between the two groups was statistically significant (*P* < 0.05). The average LPW was 8.0 ± 1.4 mm and 6.8 ± 1.6 mm in meniscus injury and non-meniscus injury groups with a significant difference (*P* < 0.05). The optimal predictive cut-off value of LPD and LPW was 7.9 mm (sensitivity-95.0%, specificity-58.8%, area under the curve (AUC-0.818) and 7.5 mm (sensitivity-70.0%, specificity - 70.6%, AUC - 0.724), respectively. The meniscus injury group mainly showed injuries involving the mid-body and posterior horn of lateral meniscus (98.1%, 157/160).

**Conclusions:**

The mid-body and posterior horn of lateral meniscus injury is more likely to occur in patients with Schatzker II TPFs when LPD > 7.9 mm and/or LPW > 7.5 mm on CT. These findings will definitely provide guidance for orthopedic surgeons in treating such injuries. During the operation, more attention is required be paid to the treatment of the meniscus and the possible fracture reduction difficulties and poor alignment caused by meniscus rupture and incarceration should be fully considered in order to achieve better surgical results.

## Background

Tibial plateau fractures (TPFs) are usually accompanied by injuries of soft tissues including the medial and lateral meniscus, medial and lateral collateral ligaments, and anterior and posterior cruciate ligaments [1]. Under these conditions, early diagnosis and treatment of meniscus and ligament injuries can often provide a better prognosis of knee function [2–4]. In clinical practice, it is noticeable that there exists a limit of physical examinations on patients due to joint pain, swelling, and confined activity. The identification of soft tissue damage often depends on imaging examinations and intraoperatively direct or arthroscopic explorations. In this regard, magnetic resonance imaging (MRI) has been confirmed to have unique advantages in the diagnosis of meniscus and ligament injuries of the knee joint [5]. However, with respect to the high cost, long examination time, and relatively delayed appointment scheduling of MRI, it is still not widely promoted and used in non-tertiary hospitals in China. According to the Chinese experts’ consensus on the diagnosis and treatment of TPFs published in 2015 [6], there is a great deal of controversy on whether routine MRI examination is needed for fresh fractures. Thus the majority of patients with TPFs receive preoperative X-ray and CT examinations. In recent years, some clinical studies have pointed out that preoperative knee X-ray and CT examination can also indicate TPFs combined with soft tissue injury, especially meniscus injury [7–13].

Several lines of evidence have suggested that Schatzker II are the most common type of TPFs. Schatzker II fractures are characterized by lateral tibial plateau cleavage with collapse of the articular surface and often associated with meniscus injury [14, 15]. The incidence of Schatzker II TPFs combined with lateral meniscus injury, has been reported to be as high as 91.0% [16]. In a considerable number of patients, it can be hypothesized that the association between Schatzker II TPFs and lateral meniscus injury might be a constant finding. Accordingly, this study aimed to analyze the frequency and patterns of lateral meniscus injury with the use of arthroscopic examination following open reduction internal fixation (ORIF) of Schatzker II TPFs. Furthermore, we conducted a preoperative morphological evaluation of lateral plateau fractures on CT images through the measurement of both lateral plateau depression (LPD) and lateral plateau widening (LPW) values for predicting the possibility of lateral meniscus injury, which could provide increasing evidence for a more comprehensive and integral treatment of bone and soft tissue injuries in Schatzker II TPFs patients.

## Methods

### General data

From August 2014 to January 2021, patients with Schatzker II TPFs were consecutively recruited from two hospital-based orthopedic departments. The hospital ethics committees approved the study. Inclusion criteria were: (1) a history of knee joint trauma with a definitive diagnosis of Schatzker II TPFs based on Schatzker classification [17] by X-ray and CT, and an articular surface collapse or separation distance of more than 3 mm; (2) time from injury to operation not exceeding 3 weeks; (3) informed patient consent. The exclusion criteria were: (1) severe bone metabolic diseases, pathological fractures, etc.; (2) periarticular fractures in ipsilateral lower limb and simple intercondylar protrusion fractures; (3) a history of tibial plateau trauma and/or lateral meniscus injury; (4) refusal of surgery for conservative treatment. A total of 296 patients with Schatzker II TPFs met the inclusion criteria and were enrolled in this study. Clinical information and medical history such as age, sex, and pre-existing medical conditions were recorded by the same doctor in each hospital.

### Surgery

All patients received preoperative X-ray and CT examinations to assess the fracture injury. At the first step, all recruited patients underwent ORIF surgery using buttress plates and screws with the objective of good reduction of the articular surface, stable fracture support and fixation, and avoidance of soft tissue complications. Exploration for lateral meniscus injury was conducted during the operation. Repairs or sutures were performed under direct vision when damage to the anterior horn and body of lateral meniscus were identified, for example rupture and incarceration. Subsequently, secondary inspection of the meniscus and ligaments using arthroscopy was undertaken to check the articular surface collapse and soft tissue response to treatment. Lateral/medial meniscus, anterior/posterior cruciate ligaments, lateral/medial collateral ligaments, etc. were routinely examined. If the posterior horn of the lateral meniscus or medial meniscus was injured and difficult to be repaired or shaped directly in the open incision, arthroscopic treatment was performed. The entire operation was completed after cleaning up the extravasated blood in the joint cavity.

### Observation index

**Fig. 1:**
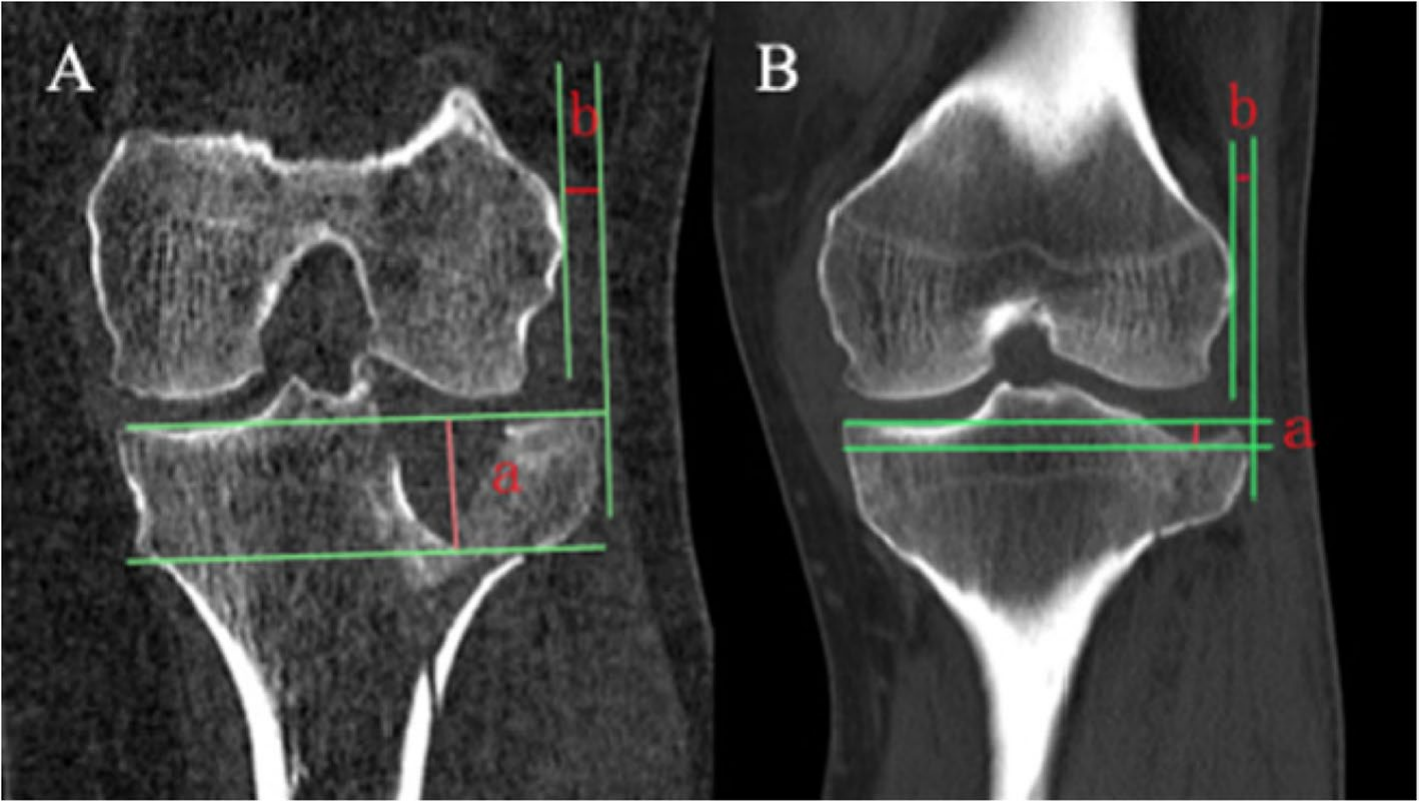
Measurement of LPD and LPW (**A**) A 41-year-old woman who had Schatzker type II tibial plateau fracture and lateral meniscus injury, a = LPD (19.2 mm), b = LPW (5.2 mm); (**B**) A 45-year-old man who had Schatzker type II tibial plateau fracture without lateral meniscus injury, a = LPD (4.1 mm), b = LPW (3.9 mm). LPD, lateral plateau depression; LPW, lateral plateau widening

Preoperative CT examination results were summarized and reviewed independently by two surgeons of more than 10 years training from each hospital who could expertly use the Picture Archiving and Communication System to evaluate the tibial lateral plateau. The surgeons did not know the patient physical examination or intraoperative exploration results. The main imaging measurement indexes were as follows: (1) LPD, which refers to the distance from the tangent line of the articular surface of the tibial plateau to the lowest point of the collapsed fracture fragment; (2) LPW, which is defined as the distance from the tangent line of the lateral femoral condyle to the farthest end of the split fracture fragment. The surgeons, who have more than 10 years’ experience as knee specialists, used the same recording standards and methods to measure LPD and LPW twice of all 296 patients within a 3-week interval. Each measurement at a specific time-point was performed three times and the average value was taken. The measurement schematic diagram is shown in Fig. 1.

### Statistical methods

All data were analyzed by SPSS 25.0 statistical software (SPSS, Chicago, IL, USA) and the G-power programs (version 3.1.5, University Düsseldorf, Germany). Kolmogorov-Smirnov test was used to distinguish whether continuous variables obeyed normal distribution. In this study, age, LPD, and LPW were all normally distributed and demonstrated homogeneity of variance, which were expressed in the form of mean ± standard deviation. In meniscus injury and non-meniscus injury patients, the numerical variables including age, LPD, and LPW were all compared by the Student’s *t* test. Patient demographics were evaluated by the Chi-square test if categorical variables. Receiver operator characteristic (ROC) curves were plotted to identify the cut-off values of both LPD and LPW for the risk of lateral meniscus injury with the maximum point of the Youden index. The cut-off value was defined according to the results of ROC curves and area under the curve (AUC) analysis.

A power analysis was performed using a proportion z-test with a one-tailed, α error established at 5% to detect differences in the incidence of lateral meniscus injury according to LPD and LPW. The calculated powers were 0.86 and 0.82 with a critical z value of 1.75 and 1.69, which were deemed acceptable. Intra- and inter-observer reliabilities were assessed with the intraclass correlation coefficient (ICC) with consistency. The observer reliability was set as poor (ICC < 0.5), moderate (0.5 < ICC < 0.75), good (0.75 < ICC < 0.9), or excellent (ICC > 0.9). For all the tests, a *P*-value < 0.05 was considered statistically significant.

## Results

During the study 296 patients with Schatzker II TPFs were recruited, of which 160 patients (54.0%) had lateral meniscus injury (including 7 patients with both medial and lateral meniscus injuries). The 160 patients (88 males and 72 females) were incorporated into meniscus injury group with an average age of 46.0 ± 16.0 years old. Thereinto, lateral meniscus, simple or combined, injury was identified in the anterior horn (*n* = 5), midbody (*n* = 114) or posterior horn (*n* = 64). The proportion of lateral meniscus injury involving the midbody or posterior horn was 97.5% (156/160) (Table 1). In addition, the average age of non-meniscus injury patients (86 males and 50 females) was 43.8 ± 15.2 years (Table 2). A parallel distribution in hypertension and diabetes could also be observed (Table 2). There was no statistically significant difference between the above comparisons in the mean age and the proportion of sex hypertension, and diabetes (*P* > 0.05) (Table 2). No cruciate ligament injury was found in all patients after second-look arthroscopy and 13 patients were complicated with medial collateral ligament injury. Among them, 9 patients received conservative treatment with adjustable knee brace after surgery, and 4 patients received the surgery of ligament repair.

**Table 1:** Different locations and patterns of lateral meniscus injury

Locations and patterns of lateral meniscus injury	No.
**Simple injury of lateral meniscus**	**130**
The anterior horn	**3**
Longitudinal/oblique tears	1
Radial tears	2
The midbody	**86**
Longitudinal/oblique tears	11
Radial tears	13
Horizontal tears	8
Meniscocapsular separation	54
The posterior horn	**41**
Longitudinal/oblique tears	7
Radial tears	6
Horizontal tears	4
Meniscocapsular separation	24
**Multiple injuries of lateral meniscus**	**23**
Radial tears in the anterior horn and midbody	1
Longitudinal/oblique tears in the midbody and posterior horn	4
Radial tears in the midbody and posterior horn	5
Horizontal tears in the midbody and posterior horn	3
Radial tears in the midbody and meniscocapsular separation in the posterior horn	4
Longitudinal/oblique tears in the midbody and meniscocapsular separation in the posterior horn	6
**Simultaneous injuries of medial and lateral meniscus**	**7**
Radial tears in the anterior horn of lateral meniscus and horizontal tears in the posterior horn of medial meniscus	1
Longitudinal/oblique tears in the midbody of lateral meniscus and radial tears in the posterior horn of medial meniscus	2
Longitudinal/oblique tears in the midbody of lateral meniscus and horizontal tears in the posterior horn of medial meniscus	1
Longitudinal/oblique tears in the midbody of lateral meniscus and the posterior horn of medial meniscus	1
Radial tears in the midbody of lateral meniscus and the posterior horn of medial meniscus	1
Radial tears in the posterior horn of lateral meniscus and the midbody of medial meniscus	1

*No.* Number

**Table 2:** Comparisons of general data and coronal CT results

	Lateral meniscus injury group	Non-lateral meniscus injury group	***P***-value
Patients, No. (%)	160 (54.1%)	136 (46%)	
Age, y (mean ± SD)	46.0 ± 16.0	43.8 ± 15.2	0.681^a^
Sex, No. (Males: Females)	88:72	86:50	0.151^b^
Hypertension, No.	40	24	0.126^b^
Diabetes, No.	16	8	0.196^b^
LPD, mm (mean ± SD)	13.2 ± 3.2	9.4 ± 3.2	< 0.001^a^
LPW, mm (mean ± SD)	8.0 ± 1.4	6.8 ± 1.6	0.017^a^

*No.* Number; *y* years; *SD* Standard deviation; *LPD* Lateral plateau depression; *LPW* Lateral plateau widening

^a^The Student’s *t*-test

^b^Pearson Chi-square test

Both the LPD and LPW had good intra- (ICC = 0.93; ICC = 0.89) and inter-observer reliabilities (ICC = 0.91; ICC = 0.87). In the meniscus injury group, the minimum, maximum, and mean ± SD LPD were 8.0 mm, 25.4 mm, 13.2 ± 3.2 mm, respectively. In the group without meniscus injury, the minimum and maximum LPD were 5.1 mm and 16.0 mm with a mean ± SD value of 9.4 ± 3.2 mm. There was a significant difference between the meniscus and non-meniscus injury groups for mean LPD (*P* < 0.001). The mean LPW were 8.0 ± 1.4 mm (minimum 2.5; maximum 28.1 mm) for the meniscus injury group and 6.8 ± 1.6 mm (minimum 1.1; maximum 27.9 mm) for the non-meniscus injury group, and the difference was statistically significant (*P* = 0.017) (Table 2).

**Fig. 2:**
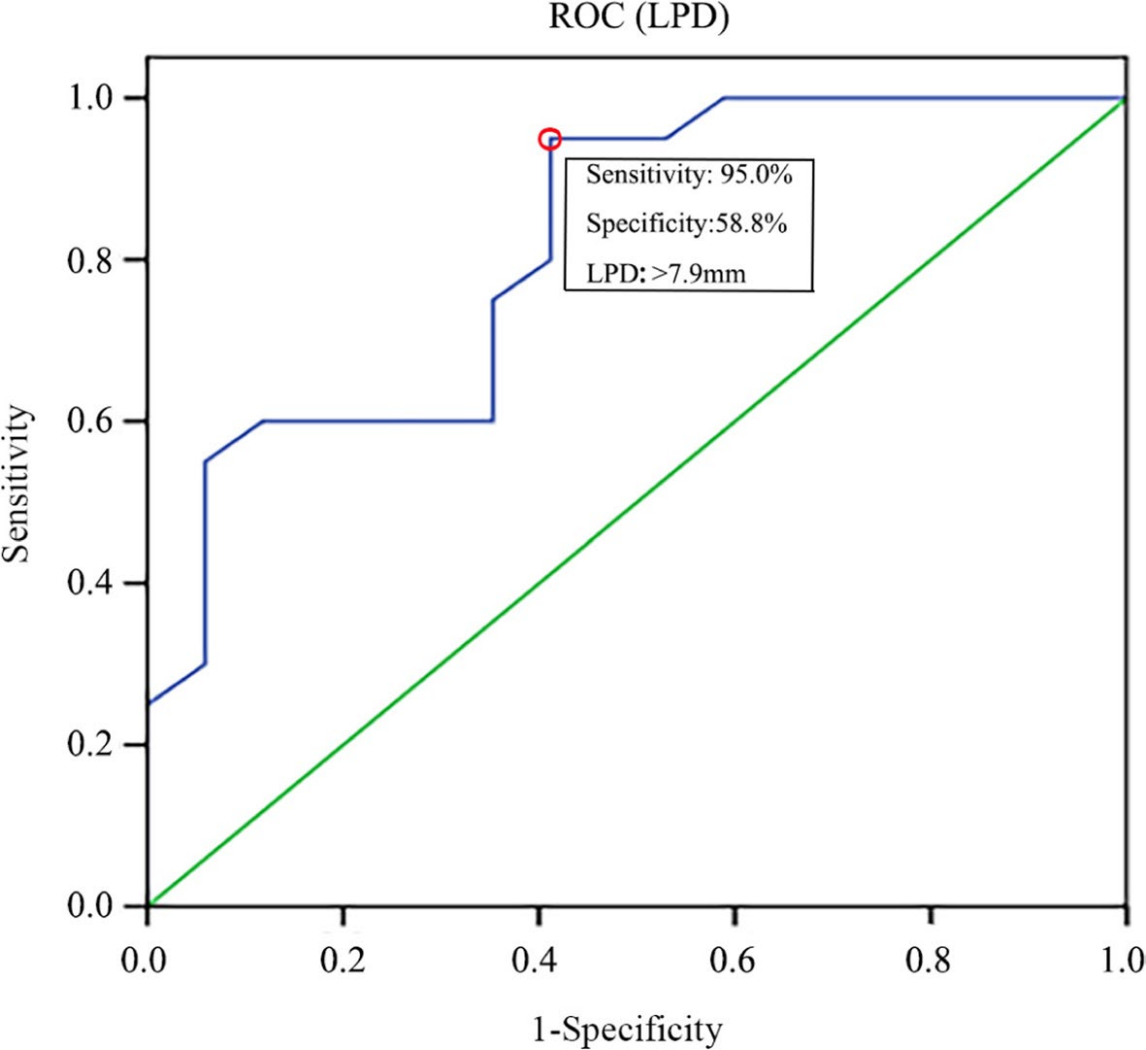
ROC analysis of LPD to predict lateral meniscus injury in Schatzker type II tibial plateau fracture patients. ROC, Receiver operating characteristic; LPD*,* lateral plateau widening

**Fig. 3:**
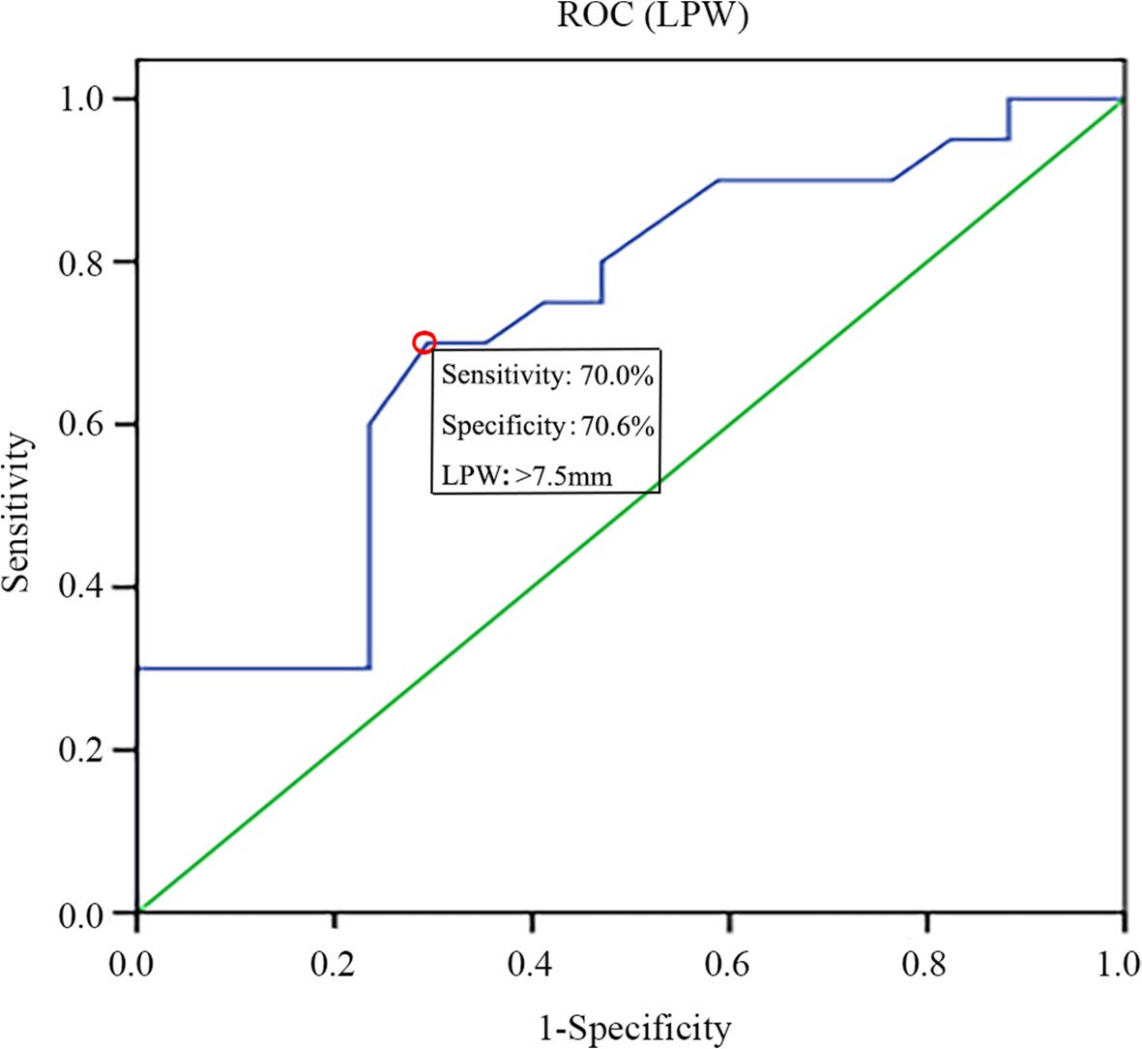
ROC analysis of LPW to predict lateral meniscus injury in Schatzker type II tibial plateau fracture patients. ROC, Receiver operating characteristic; LPW*,* lateral plateau depression

By drawing the ROC curve, it could be found that the optimal cut-off point for LPD was 7.9 mm (sensitivity - 95.0%, specificity - 58.8%, AUC - 0.818) (Fig. 2). The optimal LPW cut-off point was 7.5 mm (sensitivity - 70.0%, specificity - 70.6%, AUC - 0.724) (Fig. 3).

## Discussion

This retrospective study focused on Schatzker II TPFs. When LPD > 7.9 mm, the positive rate for the diagnosis of lateral meniscus injury was 95% with a specificity of 59%. When LPW > 7.5 mm, the positive rate for a lateral meniscus injury diagnosis was 70% with a specificity of 71%. Using arthroscopy, it was determined that 98% of lateral meniscus injuries occurred in the midbody or posterior horn and the most common pattern of meniscal tear was meniscocapsular separation.

According to relevant studies, the probability of TPFs with meniscus injury is approximately from 28.6 to 81.0% and lateral meniscus injury is dominant [7–11, 13, 18, 19]. Over the last decade or so, it is worth nothing that some clinical studies have gradually focused on the correlation between the X-ray, MRI, and CT findings of lateral plateau and the injury of lateral meniscus (Table 3). By analyzing the X-ray and MRI manifestations of lateral plateau in 62 Schatzker type II fracture patients, Gardner et al. [8] found that when lateral plateau collapsed > 6 mm and the width increased > 5 mm, the positive rate of lateral meniscus injury could reach as high as 83.0%. Ringus et al. [9] also studied the CT findings of 85 Schatzker type I-VI plateau fractures, of which 21 were type II fractures. They indicated an 8-fold increase in the risk of lateral meniscus tear when the articular surface depression was ≥10 mm. Durakbasa et al. [7] reported 20 cases of Schatzker type II plateau fracture with lateral plateau X-ray images and intraoperative direct vision of lateral meniscus, and demonstrated that a plateau depression ≥14 mm and/or widening ≥10 mm is related with a significantly high risk of meniscus injury. Furthermore, Tang et al. [13] compared the CT presentations with arthroscopic examination results of 132 patients of Schatzker I-VI plateau fractures, among which 25 cases were type II fractures. The results showed that the positive incidence of lateral meniscus injury was about 70.3% when plateau collapsed > 11 mm. Kolb et al. [11] researched CT and MRI appearances of 55 patients of Schatzker type I-III plateau fractures (50 patients of type II fractures) and proposed that the probability of lateral meniscus injury increased by 40% for each 1 mm increase in LPW. Lately, a study based on the CT and arthroscopic results of 102 patients of Schatzker I-VI plateau fractures (33 patients of type II fractures) revealed a higher risk of lateral meniscus injury in patients with > 6.3 mm of lateral joint depression [10]. Together, our findings were somewhat consistent with those of the above studies. Notwithstanding this, the differences in radiographical measurement methods of LPD and LPW as well as Schatzker types of included patients are the main reasons for the lack of relatively definitive conclusions. There is increasing evidence that MRI examination may potentially overestimate the true prevalence of meniscus injury associated with TPFs and peripheral longitudinal tear of lateral meniscus can be easily neglected [20, 21]. We insisted that a comprehensive assessment of intraoperative soft tissue injury which were confirmed by second-look arthroscopy was quite essential. Still, different sample sizes and subjective bias may also affect the results.

**Table 3:** Summary of studies on the correlation between the morphology of lateral plateau in TPFs and lateral meniscus injury

Authors	Number of patients	Schatzker type	The rate of lateral meniscus injury in TPFS	Conclusions
Durakbasa et al. [7]	20	II	60.0%	X-ray: collapse ≥14 mm, widening ≥10 mm, the positive rate of lateral meniscus injury was 100%.
Gardner et al. [8]	62	II	73.0%	X-ray: collapse > 6 mm, widening > 5 mm, the positive rate of lateral meniscus injury was 83.0%.
Ringus et al. [9]	85	I-VI	28.6%	Coronal CT: collapse > 10 mm, an 8-fold increase in the risk of lateral meniscus tear.
Chang et al. [10]	102	I-VI	63.6%	Coronal CT: collapse > 6.3 mm, the positive rate of lateral meniscus injury was 75.5%.
Kolb et al. [11]	55	I-III	34.5%	Coronal CT: per 1 mm widening, the positive rate of lateral meniscus injury increased by 40%.
Tang et al. [13]	132	I-VI	56.0%	Coronal CT: collapse > 11 mm, the positive rate of lateral meniscus injury was 70.3%.

*TPFs* Tibial plateau fractures

In Schatzker II TPFs, the morphology of lateral plateau is positively correlated with the injury of lateral meniscus. In most cases, the combination of axial loading of femoral condyle and valgus force can lead to lateral plateau splitting and collapse. After fracture, the knee joint often undergoes varus or rotation caused by the continued transmission of violence and lateral meniscus is prone to be injured under stress [18]. The ROC curves for LPD and LPW showed that the AUC values were 0.818 and 0.724, which implied the diagnostic value of these two indicators is relatively high and well confirmed that both LPD and LPW could be used as predicting factors of lateral meniscus injury. The diagnosis of lateral meniscus injury was made on the basis of intraoperatively direct exploration and patients with a history of old meniscus injury were excluded. Importantly, the average age of patients was 45 years old, which can potentially ensure that lateral meniscus injury is caused by trauma. This is in line with the purpose of this study and the conclusions are relatively accurate and reliable. Accordingly, measuring LPD and LPW values is of considerable guiding significance for the comprehensive surgical management of Schatzker II TPFs and may be a kind of technical evaluation means to make up for the lack of preoperative MRI diagnosis in hospitals below tertiary Grade in China.

For the first time, we found that lateral meniscus injury occurred in different locations with various patterns in Schatzker II TPFs patients. Meanwhile, there were combined injuries of the anterior horn, midbody, and posterior horn in the meniscus, as well as simultaneous injuries of medial and lateral meniscus. A relatively high proportion of patients with meniscocapsular separation (ie, meniscus peripheral rim tears or avulsions) (88/160, 55.0%) is also in accordance with the results of Stahl et al. [19] Intrinsically, lateral meniscus injury majorly occurred in the midbody and posterior horn, which may be deemed as a novel finding and related to the injury mechanism of such patients. The injury involving the anterior horn of lateral meniscus may be potentially caused by hyperextension and valgus knee joint during the injury process. Taking into account the concept and incidence of tibial plateau hyperextensible and valgus injury put forward by Gonzalez et al. [22], we have reasons to believe that the possibility of damaging the anterior horn of lateral meniscus in type II plateau fracture is quite low. Taken together, it is tempting to speculate that this groundbreaking finding of the locations and patterns of meniscus injury can provide a substantial basis for a relatively precise treatment on soft tissues in Schatzker II TPFs patients. Conventional follow-up exploration under arthroscopy after internal fixation of fracture can better deal with the posterior horn of lateral meniscus, medial meniscus, ligament and other injuries, so as to achieve better soft tissue repair and avoid missed diagnosis. The surgical treatment under direct vision may be mostly meniscoplasty and meniscectomy, which is more likely to cause long-term complications after surgery. Meanwhile, the incidence of simultaneous medial and lateral meniscus injury was 4.4% (7/160). The omission of medial meniscus injury during open surgery may also be an important cause of postoperative pain in patients with Schatzker II TPFs, which has been attracting much attention from the orthopedic surgeons.

Certainly, some limitations existed in our study. There would always be minor errors in artificially measuring LPD and LPW. Still, patients enrolled in this study were limited to those with Schatzker types II TPFs and the results relevant to other types of TPFs could not be ascertained. Moreover, we failed to compare the diagnostic values between MRI and CT because MRI is not routinely performed for Schatzker II TPFs patients in the hospitals where this study was conducted. The existing data on the incidence of meniscus injury associated with TPFs was reported to be in the range of 49.0–91.0% by performing preoperative MRI [16, 23, 24]. In spite of this, the emerging evidence suggested a lower incidence of meniscus injury requiring surgical intervention than previously demonstrated according to MRI images before surgery [19]. Recently, Salari et al. described that CT measurement of fracture location and articular impaction/displacement in TPFs can be used to predict lateral meniscus injury with a high accuracy. With the further expansion of the sample size, rationally analyzing CT images to thoroughly study the exact relationship between the location and pattern of meniscus injury and the morphology of tibial plateau fractures in different types will obtain more accurate referenced significance in clinic.

## Conclusions

In summary, the present study showed that the coronal CT morphology of Schatzker II TPFs was tightly correlated with the lateral meniscus injury. When LPD > 7.9 mm and/or LPW > 7.5 mm, it is extremely necessary to consider the influential impact of the injury to the midbody or posterior horn of lateral meniscus on fracture reduction and soft tissue repair during the operation. At the same time, conditionally using arthroscopy after fracture fixation will be beneficial to obtain better postoperative outcomes.

## Data Availability

The datasets generated and analyzed during the current study are not publicly available due to limitations of ethical approval involving the patient data and anonymity but are available from the corresponding author on reasonable request.

## Abbreviations

TPFs: Tibial plateau fractures
LPD: Lateral plateau depression
LPW: Lateral plateau widening
MRI: Magnetic resonance imaging
ORIF: Open reduction internal fixation
ROC: Receiver operating characteristic
AUC: Area under the curve
